# Efficacy and safety of Sultamicillin (Ampicillin/Sulbactan) and Amoxicillin/Clavulanic Acid in the treatment of upper respiratory tract infections in adults - an open-label, multicentric, randomized trial

**DOI:** 10.1016/S1808-8694(15)30041-0

**Published:** 2015-10-19

**Authors:** João Batista Ferreira, Priscila Bogar Rapoport, Eulália Sakano, Arthur Octávio De Ávila Kós, Otávio B. Piltcher, Shirley Shizue Nagata Pignatari, Sebastião Diógenes Pinheiro, Marcos Mocellin

**Affiliations:** a Assistant Professor, Head of the Otolaryngology Department – Surgery Department – Medical School of the UFG.; b PhD in Otolaryngology by the Medical School of the USP, Full Professor of Otolaryngology of the Medical School of the ABC.; c Head of the Rhinology Department of the Otolaryngology Service of the UNICAMP.; d Associate Professor of Otolaryngology by the Universidade Federal do Rio de Janeiro. Full Professor of the Federal University of Rio de Janeiro.; e Physician hired by the Otolaryngology Department of the University Hospital of Porto Alegre - RS.; f Assistant Professor of the Otolaryngology and Head and Neck Department - UNIFESP / EPM.; g PhD in Medicine. Physician from the University of the Walter Cantídio Hospital of the Medical School of Ceará.; h MSc and PhD from the Paulista School of Medicine – SP. Full Professor of the Otolaryngology Department of the University of Paraná. Medical School of the Federal University of Goiás.

**Keywords:** ampicillin, sulbactan, amoxicillin, clavulanic, infections, upper respiratory tract

## Abstract

Upper respiratory tract infections are the most common causes of medical visits in children and adults, demanding massive use of antibiotics. Bacterial resistance caused by beta-lactamase is one of the most serious problems in this matter. Sultamicillin, a double pro-drug of Ampicillin/Sulbactan, is a potent beta-lactamase inhibitor which can face this challenge.

**Aim:**

evaluate efficacy, safety and tolerability of Ampicillin/Sulbactan compared to Amoxicillin/Clavulanate in upper respiratory tract infections in adults.

**Methods:**

102 patients were enrolled and randomized to receive Ampicillin/Sulbactan or Amoxicillin/Clavulanate during 10 days. They were evaluated 10 and 30 days after treatment to learn about the therapeutic response.

**Results:**

There were no differences between the two groups respecting cure at the end of treatment (visit 2) or at the end of the study (visit 3). Cure ratio was 61.7% and 93.2% (visits 2 and 3) in the Amoxicillin/Clavulanate group compared to 64.4% and 97.4%, respectively, in Ampicillin/Sulbactan group. The adverse events ratio for the two groups was the same (p=0.940). The number of patients with diarrhea was greater in the group of patients receiving Amoxicillin/Clavulanate (70.6%) than in the group receiving Ampicillin/Sulbactan (29.4%) (p=0.0164).

**Conclusions:**

Ampicillin/Sulbactan is as safe and efficient as Amoxicillin/Clavulanate in the empiric treatment of upper respiratory infections in adults. The low occurrence of diarrhea in the group receiving Ampicillin/Sulbactan needs confirmation in other studies.

## INTRODUCTION

Upper airway infections in children and adults are the most common diseases, and also the most common reasons for seeking medical consult in primary care. They also constitute the major diagnosis that require the use of antibiotics. Its diagnosis and treatment have an impact not only from the stand point of population health, but also from the social and economical stand point, because of the cost of medical care, cost of antibiotic used and for the loss they represent as far as work and school absenteeism are concerned. Taking Acute Otitis Media in children for instance, American data points towards a total of 16 million visits on this account in the year 2000. These visits generate a total 13 million prescriptions, meaning 802 antibiotic prescribed for each group of 1,000 visits. The cost of each antibiotic therapy varied from US$ 10 to US$ 100.

The proper use of antibiotics not only in a hospital setting, but also in handling the most common infections in the community, has been the objective of ever increasing debates, in an attempt to contain the growing resistance rates that have been seen. Great emphasis have been given to standardizations published by national entities and universities, where evidence based approach reviews the major points related to the diagnosis and treatment of infections. Many standardizations have been recently published about otitis, sinusitis and pharyngo-tonsillitis^2^, ^3^, ^4^, ^5^.

The major discussions in these documents are focused on whether or not it is necessary to use antibiotics in AURTI (acute upper respiratory tract infections) and, specially in non-complicated Acute Otitis Media (AOM). The recommendation of initial use of symptomatic medication followed by antibiotics if there is no clinical improvement with the patient, is based on the high incidence of viral etiology in these pathologies. Notwithstanding, it can not be followed in many cases when the decision has to be made quickly, without the possibility of having the patient return, as is the case of ER visits. For this reason, great emphasis should be given to aspects that support the most accurate possible diagnosis. And, from then, the choice of antibiotic^2^, ^3^, ^5^.

As far as diagnosis is concerned, there is some consensus: history of acute onset of symptoms; presence of exudation/inflammation signs in the middle ear, choanae and nasal cavities or throat; pain, satellite lymph node involvement and fever.

The difficulties lie on the real possibility of establishing reliable and reproductive parameters to establish the presence of inflammatory and/or exudative clinical signs. The different clinical experiences and the available technology translate themselves into different diagnostic approaches in about 50% of the non-complicated cases. Particularly in pharyngo-tonsillitis, the germ culture and the quick strep test are recommended, however they are difficult to have in our settings^6^, ^7^, ^8^, ^9^.

AURTI etiology rests on the knowledge of the habitual flora in the upper airways and the rarely isolated prevalent micro-organisms. In pharyngo-tonsillitis the prevalent bacterial agent is the group A hemolytic β streptococcus. In AOM and sinusitis the prevalent micro-organisms are Streptococcus pneumonia, Haemophilus influenza and Moraxella catarhalis.

The choice of the antimicrobial agent should be guided by this knowledge. For clinicians and pediatricians, the choice of antimicrobial agent is a key issue. Right now the attention of the medical community and the general public is directed towards the high cost of medication and the growing rates of antibiotic resistance^10^. Therefore, judicious choice of antimicrobial agents is becoming a priority.

Ampicillin/Sulbactan is a combination of antibiotics made up of ampicillin, a betalactam and sulbactam, a beta-lactamase inhibitor^11^. By adding sulbactan we considerably broaden the ampicillin action spectrum, including B fragilis, Klebsiella sp, S aureus (MS) and H influenza.

The betalactam core destruction of penicillin and cephalosporin by bacterial b-lactamase, constitutes the clinical most important mechanism of bacterial resistance against these antibiotics. The lack of receptor binding may be caused by: (1) changes in the permeability of the cell membrane and/or (2) changes in target site biding capacity and (3) enzymatic destruction by antibiotics.

The third of these three main causes is the most frequent origin of antibiotic inefficacy. Beta-lactamase production causing bacterial resistance is one of the most severe problems of clinical practice today. B-lactamase may be produced either by gram positive or by gram negative bacteria. They destroy the antibiotic Betalactam core, turning them useless. This destruction occurs by the hydrolysis of the amide bond in the betalactam ring, resulting in the production of acid derivatives that do not have antibacterial properties. Clinical failure appears when a sufficient number of antibiotic molecules is neutralized^12^, ^13^.

Unasynâ (sultamicillin) is an antibiotic developed specifically to face these problems^14^, ^15^. It is an ampicilin and sulbactam double pro-drug, a powerful beta-lactamase inhibitor. The ampicillin/sulbactam mechanism of action depends mainly on the sulbactam bond, a suicide inhibitor, with a large number of b-lactamase molecules. Sulbactam hydrolyzed fragments remain irreversibly bound to this enzyme, thus forming an inactive complex.

Ampicillin/Sulbactam pharmacokinetic studies have shown that the inactive pro-drug is well absorbed in the intestine. Ampicillin/Sulbactam is hydrolyzed in two compounds in the intestine wall, sulbactam and ampicillin. The total ampicillin distributed is approximately twice larger when compared to the oral distribution of ampicillin alone. The high serum level achieved is followed by optimum ampicillin and sulbactam tissue penetration. And finally, the clearance route for both active drugs is the urine^16^, ^17^, ^18^.

The Ampicillin/Sulbactam action spectrum encompasses gram positive coccus and rods, aerobic gram negative and some anaerobes, namely:

Peptococcus and Peptostreptococcus spp, anaerobes, Group B Streptococcus, Enterococcus faecalis, Streptococcus pneumoniae, Streptococcus pyogenes, Streptococcus viridans (alpha-hemolytic), C diphtheriae, Clostridium spp (Cl tetani, Cl perfringens, Cl botulinum), Listeria monocytogenes, Haemophilus influenza, E coli (over 50% of the strains may be resistant), P mirabilis, Salmonella spp (S typhimurium may be 30% to 40% resistant), Shigella flexneri, Fusobacterium spp.

The Ampicillin-Sulbactam combination (Sultamicillin) shows marked synergy. The MIC of many ampicillinresistant pathogens is reduced, being very similar to those sensitive organisms of the same species. The ampicillin activity against sensitive organisms remains the same^19^, ^20^.

On the other hand, the production of significant quantities of b-lactamase have been associated to cephalosporin resistance (Cephoxytin is the most powerful resistance inducer) and to the ureidopenicillins. Incubation with sub lethal concentrations of sulbactam clavulanic acid and numerous betalactam antibiotics has shown that sulbactam did not induce measurable levels of chromosome betalactam, while clavulanic acid induced 30% in all the strains studied.

Therefore, clavulanic acid may cause significant induction. This fact is in agreement with the results of an in vitro study on the negative R factor of the carbenicillinresistant P. aeruginosa strains which suggests the existence of an antagonism between the clavulanic acid and azlocillin combination, while adding sulbactam does not seem to affect the penicillin minimum inhibitory concentrations. So far, no antagonism between sulbactan and other betalactam antibiotics has been observed^21^, ^22^, ^23^.

The objective of this trial was to assess Sultamicillin efficacy, safety and tolerability compared to that of Amoxicillin/Clavulanic acid, in the treatment of community acquired infections of the upper respiratory tract, specially otitis, sinusitis and pharyngo-tonsillitis.

## METHODS

Eight Brazilian research centers participated in this study, recruiting 102 patients from March 22, 2002 to July 04, 2003. The protocol design established a comparative, multicentric, open and randomized trial of Ampicillin/Sulbactan compared to Amoxicillin/Clavulanic acid. Patients were given 375mg bid PO Ampicillin/Sulbactan or 500mg tid PO Amoxicillin/Clavulanic acid after randomization. The study included 3 medical visits and lasted 34 days, having one initial visit (V-1), followed by a treatment period of 10 consecutive days, and 2 later follow up visits between days 10 and 13 (V-2 – end of treatment) and between days 26 and 34 (V-3 – end of trial).

On visit 1, male and female patients older than 12 years and weighing 30 Kg and more were diagnosed as carriers of bacterial infections in the ears, nose and/or throat, concomitant or not, through clinical history and physical exam data. Inclusion criteria were defined as follows:

• Having at least 3 of the following signs/symptoms for sinusitis patients: bi-phased disease, defined as the presence of two phases of the current disease; purulent anterior or posterior rhinorrhea; purulent secretion in the nasal cavity; facial/maxillary or teeth pain; OR
• Having at least 3 of the following signs/symptoms for otitis patients: local pain, fever of 38°C or more; otoscopy findings such as tympanic hyperemia; tympanic bulging; purulent or mucous-purulent secretion in the middle ear; OR
• Having at least 4 of the following signs/symptoms for pharyngo-tonsillitis patients: sudden onset, intense pain on swallowing; tonsillar enlargement; tonsillar hyperemia; exudate on the tonsils; painful anterior cervical lymph node disease; fever of 38°C or more.

Patients were excluded when mentioned: hypersensitivity to the trial drugs or to the betalactamic; pregnancy, breast feeding or pregnancy possibility during the trial; specific systemic disease or other medical conditions, including viral and urinary infections and meningitis that could interfere in the therapeutic response assessment, in the absorption of medications, or safety of the trial drug; treatment with any systemic antibacterial drug within 14 days before the randomization; concomitant participation in any other investigational drug clinical trial; blood donation or blood derivatives for transfusion in the 30 days prior to the treatment with the trial drugs at any time during the trial or after 30 days of the trial end; treatment with halopurinol, metrotrexate, probenecide and disulfiram, or other medications that may interfere on the trial drug assessment, and, transaminase values (ALT/SGPT or AST/SGOT) above 3 fold the upper limit of normality (ULN), and serum creatinin above 2 times the ULN.

On visits 1, 2 and 3, the patients underwent a material collection for safety exams (Complete blood count, biochemistry and pregnancy test, when indicated) and general clinical evaluation. Besides all of this, the patients underwent specific evaluation for signs and symptoms related to upper respiratory tract infections, which include the same aspects used to confirm the diagnosis.

### Evaluation of the clinical response

Efficacy was evaluated based on the clinical response seen on visits 2 and 3 classified by the investigator according to the following criteria:

Cure: absence of all signs and symptoms of the disease being studied, described in the inclusion criteria session.

Improvement: presence of at least one of the studied disease signs and symptoms described in the inclusion criteria, without the need to prescribe another antibiotic agent.

Failure: Persistence of one or more signs or symptoms of the studied disease described in the inclusion criteria, or the appearance of new signs and symptoms of the studied disease and/or the need to add another antibiotic agent or to change the study therapy.

### Safety

All adverse events seen or reported were registered with specifications of the date of onset, the duration, the severity, the evolution and the possible relation with the trial drug. Altered findings in the lab exams were also reported as adverse effects.

### Statistical analysis

The safety population was formed by 97 patients that took at least one dose of the trial medication. The ITT (intention to treat) efficacy population was made up by the patients who were given at least one dose of the assigned treatment and had at least one subsequent classification of clinical response to the instituted therapy. In other words, it was made up of 92 patients: all the 97 safety population patients minus the 5 patients who had unknown clinical response on visits 2 and 3. The efficacy PP population is made up of the patients who received at least 6 doses of the assigned treatment and had at least one subsequent clinical response to therapy classification. In this study, population PP is formed by the same ITT population patients, because all had at least 6 doses of the prescribed medication.

For clinical response we created a confidence interval of 95% for the difference in cure and cure + improvement ratio among the treatment modalities. In order to compare the average of vital signs along the visits we used the Variance Analysis with Repeated Measures, using the non-structured co-variance matrix in order to model the existing correlation among the visits of a same patient. This technique takes into account the incomplete observations and, therefore, does not rule out the patient as a whole if he/she does not have all the observations in all the visits. All the aforementioned analysis require data normality assumption which was verified and confirmed in all cases. Statistical analysis was made by the SAS version 8 software. The adopted significance level was of 0.05.

## RESULTS

### Patient traits

Eight Brazilian centers recruited 102 patients for the trial. Of these, ninety seven started the trial and received at least one dose of the medication. Only 83 patients completed the 30 days of the trial. The reasons why 14 patients were taken off the trial are as follows: adverse events - 5 patients; removal from the post-informed consent - 3 patients; loss of follow up - 3 patients; and protocol violation - 2 patients. Patient 107 was taken off because he did not return for visit 3.

Table 1 depicts the traits of the 97 security population patients at the trial onset. There was no statistically significant difference among the demographic and clinical traits of the patients belonging to the two groups.

**Table 1:** Patient characteristics in the beginning of the study: age, gender and race.

Characteristic	Amoxicillin/Clavulanic acid		Ampicillin/Sulbactan	
	N	%	N	%
Age	49	100,0	48	100,0
< 18 years	7	14,3	6	12,5
18-44 years	33	67,3	37	77,0
45-64 years	8	16,4	5	10,5
> 65 years	1	2	0	0

Race	49	100,0	48	100,0

White	39	79,6	41	85,4
Black	8	16,3	4	8,3
Yellow	2	4,1	2	4,2
Mixed races	0	0	1	2,1

Gender	49	100,0	48	100,0

Male	19	38,8	18	37,5
Female	30	61,2	30	62,5

Time of infection (days)	49	100,0	48	100,0

1 a 2 days	9	18.4	13	27.0
3 a 5 days	25	51.0	20	41.7
6 a 10 days	7	14.3	9	18.7
> 10 days	8	16.3	6	12.6

The infection time, in other words, the time span between symptoms onset and diagnosis also did not show significant difference between the groups, varying from 1 to 40 days with a 4 day median in both groups (Table 1). The type of infection presented by patients varied in both groups, although it was not significant. The Amoxicillin/Clavulanic acid group had a greater incidence of sinusitis and otitis whilst the Ampicilin/Sulbactan group patients had a larger number of pharyngo-tonsillitis.

Table 2 lists the signs and symptoms that were part of diagnostic criteria for each type of infection. Table 3 depicts signs and symptoms of patients along the trial, that is, at diagnosis – visit 1 – and their development in visits 2 and 3.

**Table 2:** Type of infection; and signs and symptoms at diagnosis

Type of infection and symptoms at diagnosis	Treatment Group			
Sinusitis	Amoxicillin/Clavulanic acid (N=20)		Ampicillin/Sulbactan (N=16)	
	N	%	N	%

Bi-phased disease	13	65.00	9	56.25
Purulent anterior or posterior rhinorrhea	20	100.00	16	100.00
Purulent secretion in nasal cavities	20	100.00	15	93.75
Facial, maxillary and teeth pain	16	80.00	16	100.00

Otitis	Amoxicillin/Clavulanic acid (N=9)		Ampicillin/Sulbactan (N=5)	
	N	%	N	%

Local pain	9	100.00	5	100.00
Fever > 38º C	7	77.78	4	80.00
Tympanic membrane hyperemia	9	100.00	5	100.00
Bulging of the tympanic membrane	7	77.78	4	80.00
Mucus-purulent secretion in the middle year	7	77.78	2	40.00

Pharyngo-tonsillitis	Amoxicillin/Clavulanic acid (N=20)		Ampicillin/Sulbactan (N=26)	
	N	%	N	%

Sudden onset, intense swallowing pain	19	95.00	24	92.31
Tonsil enlargement	19	95.00	21	80.77
Tonsil hyperemia	20	100.00	26	100.00
Presence of tonsil exudate	20	100.00	25	96.15
Anterior painful neck lymph node disease	18	90.00	22	84.62
Fever > 38º C	15	75.00	20	76.92

**Table 3:** Proportions (%) of patients who presented one of the following signs/symptoms along the study, according to treatment group

	Amoxicillin-clavulanic acid			Ampicillin/Sulbactan		
Sign/Symptom	V1	V2	V3	V1	V2	V3
Headache	59	9	0	81	2	14
Irritability	31	6	3	38	0	2
Lethargy	41	6	0	60	2	5
Post-nasal drip	37	6	0	42	0	18
Purulent rhinorrhea	43	4	0	38	0	9
Nasal mucosa edema	53	23	8	48	2	23
Obstructive nasal secretion, epistaxis, cacosmia	37	4	0	35	0	2
Fontal and maxillary sinuses tenderness	41	2	0	38	2	7
Ear pain	24	4	0	29	2	2
Hyperemic or bulged tympanic membrane	20	6	0	17	5	0
Middle year mucous-purulent secretion	33	15	0	5	11	0
Swallowing pain	51	2	0	67	0	0
Pharynx hyperemia or edema	57	11	3	75	0	9
Tonsillar exudate	41	2	0	54	0	2
Painful anterior neck lymph node disease	64	16	0	77	0	7
Fever	63	4	0	65	0	0
Cough	35	4	0	46	2	11
Halitosis	45	9	3	67	0	11
Others	61	19	2	47	23	0

V1: Visit 1 – Study onset

V2: Visit 2 – Treatment end

V3: Visit 3 – Trial end

### Efficacy and clinical answer

There were no evidences of differences between the two groups in regards to the proportion of patients who did not show cure at treatment end (visit 2) or at trial end (visit 3). In the Amoxicillin/Clavulanic acid group, cure rates were 61.7% and 93.2% on visits 2 and 3, respectively. In the Ampicilin/Sulbactan group, cure rates were 64.4% and 97.4%, respectively. Table 4 depicts Confidence intervals for the difference among cure ratios for both groups, as well as the results of the Fisher exact test. Four patients had treatment failure on the third visit after having reported cure on the second visit. Of these, 3 were from the Amoxicillin/Clavulanic acid and 1 from the Ampicillin/Sulbactan treated group.

**Table 4:** Ratio of cured patients at treatment end (V2) and at trial end (V3).

Cure ratio	Amoxicillin/Clavulanic acid	Ampicillin/Sulbactan	Difference(Sult - Amox)	95% CI for the difference	Exact Fisher Test
Visit 2	61.70	64.44	2.74	[ -16.97; 22.46 ]	P=0.9327
Visit 3	93.18	97.44	4.26	[ -4.69; 13.20 ]	P=0.6185

### Adverse Effects

There were 56 adverse effects along the trial (30 in the Amoxicillin/Clavulanic acid group and 26 in the Ampicillin/Sulbactan group). The total number of patients who had adverse effects was 34, representing 35% of the total number of participants in the trial. Of these, 17 belonged to the Amoxicillin-Clavulanic acid group and 17 to the Ampicillin/Sulbactan group. The ratio of patients who experience at least one adverse effect was similar in both groups (p = 0.940). Notwithstanding, among the 34 patients who had adverse effects, the ratio of patients with diarrhea was significant higher in the Amoxicillin-Clavulanic acid group (70.6%) when compared to the Ampicilin/Sulbactan (29.4%) group, with p = 0.0164.

**Table 5:** Adverse effect of which causality was not ruled out by the investigator.

Type of adverse effect	Amoxicillin/Clavulanic acid	Ampicillin/Sulbactan
Abdominal pain	1	2
Epigastric pain	1	0
Anemia	1	1
Elevated TGO	0	1
Elevated Creatinin	1	0
Diarrhea	12	4
Dysphonia	0	1
Borborygmy	1	1
Irritability	0	1
Nausea	1	2
Sinusitis	0	1
Vaginal candidiasis	1	0
Vomit	2	0

As to laboratory work up, no relevant alterations were found after the use of trial medications. The altered values were already present on visit 1 for both groups.

According to protocol criteria, there was no severe adverse effect. Only 5 patients were removed from the trial because of adverse events (2 on the Ampicillin/Sulbactan group and 3 on the Amoxicillin-Clavulanic acid group). The 9 adverse effects of these 5 patients were: diarrhea – 3 Amoxicillin-Clavulanic acid related events and one Ampicilin/Sulbactan-related event; lethargy – 1 Amoxicillin-Clavulanic-acid-related event; irritability – 1 case; worsening of tonsillitis – 1 case; worsening of sinusitis – 1 case; and abdominal pain – 1 case. Bellow we listed the adverse effects with cause related to the drugs used. 5 Ampicillin/Sulbactan group patients had diarrhea, however only 4 are listed here because the investigator considered that one was not related to the trial drug.

### Concomitant medication

Sixty patients (61.8%) received at least one concomitant medication. Of these, 29 belonged to the Amoxicillin/Clavulanic acid group and 31 to the Ampicillin/Sulbactan group. Among the drugs used, the most frequent were Dipirone and paracetamol, and there was no difference in its use among the two treatment groups.

## DISCUSSION

The results of this trial show the comparable efficacy of Ampicillin/Sulbactan and Amoxicillin/Clavulanic acid combinations in the treatment of acute upper respiratory tract infections in adults. Such results correlate to literature reports on the use of Ampicillin/Sulbactan combination in the treatment of both upper and lower airway infections^24^, ^25^, ^26^.

There was no significant difference in the proportion of patients who were cured at the end of treatment and at the end of trial in both groups. In the Ampicillin/Sulbactan group, the cure ratios increased from 64.4%, at treatment end, to 97.4% by trial end. These results lead us to conclude that, although we are facing empirical treatment, because we do not know the true AURTI etiology, Ampicillin/Sulbactan is one efficacious drug against the major bacteria that cause these infections in man. On the other hand, as far as diagnosis is concerned, the trial design allowed us to check the accuracy of the adopted criteria which are very similar to the signs and symptoms observed on clinical visits 2 and 3.

Similar studies were carried out comparing the efficacy of Ampicillin/Sulbactan and 2nd generation Cephalosporin – Cephoxytin and Cefuroxim in in-patients with pneumonias^27^, ^28^. In both groups, the results are comparable as far as efficacy and safety are concerned. In all these trials, clinical cure walked hand-in-hand with the bacteriological cure and S pneumoniae was the most frequently found micro-organism. Other trials show the comparative performance of Sultamicillin with amoxicillin alone or in combination with Clavulanic Acid in lower respiratory tract infections. The clinical response was, once again, comparable with efficacy rates varying between 84% and 94%^29^. Considering the treatment of otitis in children, Ampicillin/Sulbactan proved to be efficient, with cure rates above those seen with the use of Cefaclor^24^. From the safety stand point, our study confirms literature reports that Ampicillin/Sulbactan is a well tolerated drug. There were no severe adverse effects, and in those that really happened, both groups had similar distribution. Only diarrhea was significantly higher in the Amoxicillin/Clavulanic acid group (p=0.016). Gastrointestinal symptoms seem to be the most frequent adverse effect related to the use of Ampicillin/Sulbactan. In the same way, these symptoms and, diarrhea most specifically, are very common with the use of Amoxicillin/Clavulanic acid, making its use rather difficult sometimes, specially by children. In some measure, our data may have pointed towards a greater tolerability of Ampicillin/Sulbactan in comparison to Amoxicillin/Clavulanic acid, according to what has been reported in the literature^30^. Other studies must follow in order to investigate these aspects, with more cases and different populations.

In short, we have concluded that Ampicilin/Sulbactan is as efficient and safe as Amoxicillin/Clavulanic acid in the empirical treatment of adult acute upper respiratory tract infections, thus representing an important therapeutic alternative. This lower occurrence of diarrhea in the Ampicillin/Sulbactan group that has been observed requires further investigations.

## THANKS

This investigation was financed with resources from Pfizer Pharmaceuticals.
